# Evaluation of potentially toxic elements and ecological risks associated with environmental liabilities in Tacna, Peru

**DOI:** 10.1371/journal.pone.0311470

**Published:** 2025-11-25

**Authors:** César Julio Cáceda Quiroz, Gisela July Maraza Choque, Gabriela de Lourdes Fora Quispe, Diana Galeska Farfan Pajuelo, Edwin Denis Obando Velarde, Fulvia Chiampo, Milena Carpio Mamani

**Affiliations:** 1 Project of Bioremediation, Jorge Basadre Grohmann National University, Faculty of Sciences, Professional School of Biology-Microbiology, Tacna, Perú; 2 Department of Applied Science and Technology, Politecnico di Torino, Corso Duca degli Abruzzi, Torino, Italy; National Autonomous University of Mexico Institute of Geophysics: Universidad Nacional Autonoma de Mexico Instituto de Geofisica, MEXICO

## Abstract

Mining environmental liabilities (MELs) are abandoned deposits resulting from extractive activities that pose a high risk of contamination and remain an unresolved challenge for authorities worldwide. This study evaluated the contamination levels of potentially toxic elements (PTEs) and their associated ecological risks in MELs, using multiple environmental indices. Analyses were performed following the EPA 6020A method with acid digestion and inductively coupled plasma mass spectrometry (ICP-MS), while free cyanide and hexavalent chromium were determined using the EPA 9013A and EPA 7199 methods, respectively. The results revealed elevated concentrations of arsenic (1,102 mg/kg), cadmium (271 mg/kg), lead (15,961 mg/kg), and free cyanide (64 mg/kg), which exceeded regulatory standards by a considerable margin. Statistically significant differences were observed across the sites (p < 0.05). In addition, the presence of flora, fauna, rivers, and rural communities in proximity to these abandoned mining sites amplifies both ecological and social risks. The applied indices consistently indicated severe levels of contamination and high ecological risk across all areas evaluated. Although no statistically significant differences were found in some indices (p > 0.05), the magnitude of the recorded values remains ecologically relevant. Notably, each index has its own interpretative scale, allowing for an independent and robust evaluation of contamination severity and its potential ecological implications. Principal Component Analysis (PCA) revealed multiple sources of pollution, while Spearman correlation analysis identified strong associations among PTEs, suggesting common environmental dispersion pathways. This research provides a critical preliminary assessment of the risks associated with these environmental legacies and emphasizes the urgent need for remediation efforts at both local and global scales. The current lack of action is largely attributed to the absence of comprehensive baseline assessments. The findings underscore the importance of prioritizing their management through sustainable strategies and international policies to mitigate environmental impacts.

## Introduction

Mining is one of the oldest and most significant anthropogenic activities worldwide and remains a fundamental economic sector for many countries. However, the inadequate management of waste generated during mining activities has triggered serious environmental impacts due to the accumulation of hazardous residues and other potentially toxic elements (PTEs) [1–3]. However, inadequate waste management during mining activities has resulted in negative effects due to the accumulation of hazardous waste [4], including heavy metals (HMs) and other potentially toxic elements (PTEs). These pollutants are characterized by high toxicity, persistence in the environment, bioaccumulation potential, and their capacity to disrupt biogeochemical cycles and reduce biodiversity. They are highly toxic when interacting with environmental elements such as water, soil, and air, and have the ability to accumulate in the human body [5,6]. Living organisms can be exposed to PTEs through trophic transfer processes in food chains, where contaminants migrate from soils to plants and, subsequently, to higher organisms (soil > plants > humans or soil > plants > animals > humans) [7].

This situation has led to sociopolitical conflicts and significant public health repercussions [1]. Historically, areas affected by mining activities lacked specific measures aimed at environmental remediation, primarily due to the absence of environmental regulations and legal frameworks. Only a limited number of countries implemented mining regulations and governmental recovery programs, resulting in the global accumulation of Mining Environmental Liabilities (MELs) [3,8,9]. MELs are defined as abandoned or inactive facilities, effluents, emissions, residues, or waste deposits produced by mining operations, which pose a permanent and potential risk to public health and ecosystems [10–12].

It is estimated that more than one million abandoned mines exist worldwide, including underground galleries, alluvial mines [8], tailings, unused pits, and altered topography [13], all of which generate toxic waste [8,13]. Latin America hosts some of the world’s largest and oldest mines, particularly in countries such as Mexico, Chile, Bolivia, Peru, Colombia, and Ecuador [14]. The continent is especially vulnerable to PTE contamination due to (a) its history of intensive mining, (b) inadequate management of mining environmental liabilities, (c) weak enforcement of environmental regulations, (d) a high concentration of mining activities, and (f) the absence of biomonitoring systems [14,15]. In these regions, over 1,266 Mining Environmental Liabilities (MELs) have been officially reported in the Plurinational State of Bolivia, 492 in Chile, 522 in Colombia [9,16], and 6,026 in Peru [17]. Currently, Peru has approximately 11,743 mining concessions covering about 7,707,162 hectares of its national territory, including areas in Amazonas, Áncash, Apurímac, Arequipa, Ayacucho, Cajamarca, Cusco, Huancavelica, Huánuco, Ica, Junín, La Libertad, Lambayeque, Lima and Callao, Loreto, Madre de Dios, Moquegua, Pasco, Piura, Puno, San Martín, and Tacna [18]. Like other countries, Peru faces significant challenges regarding Mining Environmental Liabilities (MELs). Although it was the first country in Latin America to establish a legal framework for MEL sites in 1993 [19], a substantial number of these MELs originated prior to the regulation, when the standards were less stringent than they are today [20].

Environmental contamination caused by PTEs represents a significant challenge due to their high toxicity, long persistence, and non-biodegradable nature [20,21]. These elements tend to spread across agricultural fields, vegetation, and forest landscapes [22]. They also alter the biological and physicochemical properties of soils, causing detrimental effects on various plant physiological processes [23], and increasing the risk of surface [24], and groundwater contamination [25,26], as well as ecosystem degradation [27]. Furthermore, most PTEs possess carcinogenic properties [8,28], with As, Cd, Cr, and Ni classified as Category 1 heavy metals for carcinogenicity by the International Agency for Research on Cancer [28]. In Russia, high concentrations of Zn, Ni, Pb, Cu, and Cd are exacerbated by the interaction of mining waste, atmospheric precipitation, and acid mine drainage [29,30]. In Spain and China, the dispersion of these elements has contaminated surrounding areas, agricultural fields, urban soils, and forested landscapes [31,32]. In Ecuador, tailings deposits pose risks to both human populations and ecosystems due to the potential contamination of rivers, which serve as water sources for nearby communities [24]. In Peru, high concentrations of toxic elements such as Pb, As, Zn, and Cd, resulting from leaching processes, have impacted watersheds [1].

In many countries, these events generate ongoing environmental concerns, as PTEs are associated with environmental factors that can mobilize metals and residues [33,34], posing significant ecological risks. Consequently, hazard identification and risk characterization [35] are essential to assess the extent of contamination [36]. Various indices are used to evaluate the presence and concentration of anthropogenic contaminants in the environment, such as the Geoaccumulation Index (I-geo), Contamination Factor (C*f*), Pollution Index (PI) [37], Pollution Load Index (PLI), Degree of Contamination (*Cdeg*) [38], and Ecological Risk Factor (RI) [39]. Given the complexity of landscapes and surroundings, integrating multiple assessment methods becomes imperative to determine the contamination levels caused by PTEs.

In this context, it is crucial to conduct a comprehensive assessment of contamination levels and the environmental risks associated with MELs, particularly in regions like Latin America, where their environmental, social, and public health impacts are significant. This research integrates multiple environmental assessment indices to provide a more comprehensive analysis of contamination. Additionally, it focuses on characterizing potential ecological risks and designing mitigation strategies, serving as a practical tool for environmental management and decision-making. The findings are vital for strengthening remediation policies and promoting more sustainable practices in mining activities, ultimately improving the health and well-being of affected communities.

The objective of this study was to evaluate the contamination levels caused by potentially toxic elements (PTEs) generated by mining environmental liabilities (MELs) and analyze associated environmental risks using evaluation indices such as the Geoaccumulation Index, Contamination Factor, Pollution Load Index, Degree of Contamination, Ecological Risk Factor, and Potential Ecological Risk Index. This approach addressed the lack of systematic evaluations in these areas, provided essential data that enhanced the understanding of environmental impacts and served as a foundation for planning and implementing bioremediation projects aimed at mitigating soil and water contamination and containing associated environmental risks.

## Materials and methods

### Study area description

The Tacna region, located on the southern coast of Peru, is prominent in the mining sector, accounting for 6.9% of the country’s copper reserves and ranking fourth nationally. It currently reports approximately 100 mining environmental liabilities (MELs) [40], with the main subtypes identified as mine entrances, mining waste, tailings, and infrastructure [16].

**Fig 1:**
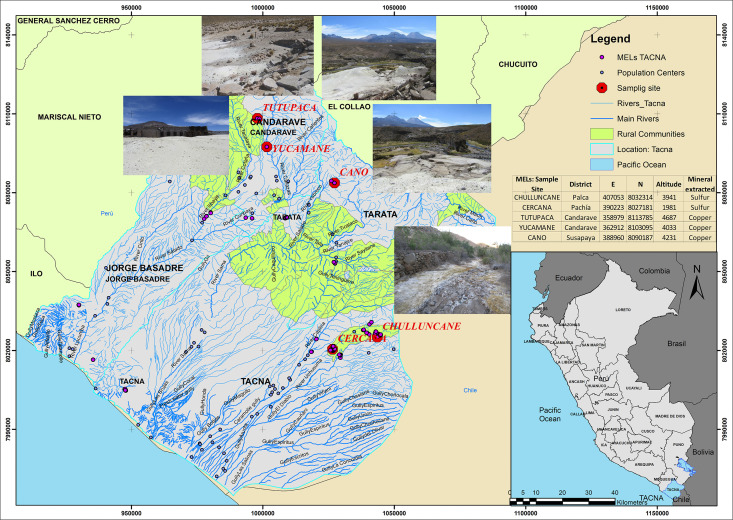
Location of Mining Environmental Liabilities (MELs) and Sampling Sites in the Tacna Region, Peru. The map was generated using QGIS 3.32 and shows the distribution of MELs across the districts of Palca, Pachía, Candarave, and Susapaya. Sampling sites include Tutupaca, Yucamane, Cano, Chulluncane, and Cercana.

The study area corresponds to the MELs located in the districts of Palca, Pachía, Candarave, and Susapaya in the Tacna region (Fig 1), which originated from former mining operations primarily focused on copper and sulfur extraction. The generation of mining tailings, combined with the absence of an identified responsible party, has led to significant environmental degradation. In addition, the region’s complex physiography and highly variable climatic conditions contribute to the intermittent activation of nearby rivers, posing a risk to surrounding ecosystems.

To assess the current condition of these MELs, a set of site-specific and environmental criteria was applied, including geolocation, associated mining activity, type of waste, distance to water bodies (0–100 meters), presence of vegetation, signs of fauna, proximity to affected sites (0–100 meters), populated centers, and rural communities. These criteria provided a comprehensive overview of the potential risks and the urgent need for remediation actions in this specific area [41].

### Sample Collection

In each study area, purposive sampling was conducted to select locations with the highest likelihood of contamination by potentially toxic elements (PTEs) [41,42]. Strategic points were chosen near water bodies, populated centers, and rural communities to adequately represent zones at greater environmental risk [41,43,44].

Sampling was carried out at five mining environmental liability (MEL) sites —Tutupaca, Yucamane, Cano, Chulluncane, and Cercana—along with one reference site representing native soil conditions [2]. A total of 20 soil samples were analyzed: five replicates were collected at the reference site, and three replicates at each of the five MEL sites [45,46]. The difference in the number of replicates between sites was due to logistical and budgetary constraints commonly encountered in field studies of mining environmental liabilities.

Approximately 500 g of surface material (0–10 cm depth) [47], was collected at each sampling point, sealed in polyethylene bags [2], and transported to the laboratory for inorganic analysis following the Peruvian soil quality standards D.S. N° 11–2017-MINAM for industrial soils [10]. The GPS coordinates of all sampling locations are presented in Fig 1.

### Concentration of metals and free cyanide in mining environmental liabilities

The concentration of the following elements was determined in a laboratory accredited by the National Quality Institute (INACAL). For the analysis of arsenic, barium, cadmium, lead, chromium, and mercury, the EPA 6020A method of Microwave-Assisted Acid Digestion of Sediments, Sludges, Soils, and Oils was used, employing Inductively Coupled Plasma Mass Spectrometry (ICP-MS) [48] with detection limits of 0.02 mg/kg, 0.04 mg/kg, 0.02 mg/kg, 0.02 mg/kg, 0.2 mg/kg, and 0.03 mg/kg, respectively. Free cyanide was analyzed following the EPA 9013A/SMEWW-APHA-AWWA-WEF Part 4120 B method [49] with a detection limit of 0.05 mg/kg, and hexavalent chromium Cr(VI) was measured using the EPA 7199.1996 method for determination of Cr(VI) by Ion Chromatography [50], with a detection limit of 0.04 mg/kg. Additionally, the pH of the samples was determined according to the US EPA 9045D method [51] using a pH meter (Hanna Instruments HI3222) and electrode (Hanna Instruments HI1043-B), with a detection limit of 0.001 pH.

### Evaluation of potential environmental risks

For the assessment of potential ecological risks and pollution levels, the following indices were determined: Geoaccumulation Index (I-geo) [52–54], Pollution Load Index [38], Pollution Index, Contamination Degree [55], Modified Contamination Degree, Ecological Risk Factor, and Potential Ecological Risk Index [38]. These indices are crucial and widely employed to evaluate contamination levels in soil and sediments, serving as valuable tools in establishing potential ecological risks [55–60].

### Geoaccumulation index (I-geo) for assessing contamination from mining environmental liabilities

The Geoaccumulation Index (I-geo) was employed to evaluate the contamination levels of each metal on the soil surface. This index takes into account both the background value and analyzes the degree of contamination. The *I-geo* was calculated as described by Mueller [55]:


$$I-geo={log}_{\text{2}}\left[\frac{C_{n}}{\text{1}.\text{5}\times B_{n}}\right]$$ (1)

Where $C_{n}$ is the concentration of the examined metal in the soil sample, $B_{n}$ is the background value, and 1.5 as a correction factor to account for potential variations in specific metal background values.

According to Mueller (1986), I-geo values are classified into seven groups: *I – geo *≤ 0 non-contaminated; 0 ≤ *I-geo *≤ 1: non-contaminated to moderately contaminated; 1 ≤ *I-geo ≤ *2: moderately contaminated; 2 < *I-geo* ≤ 3: moderately to heavily contaminated; 3 ≤ *I-geo *≤ 4: highly contaminated; 4 ≤ *I-geo* ≤ 5: heavily to extremely contaminated; 5 < *I-geo*: extremely high contamination levels.

### Pollution factor

The Pollution Factor (*C*_*f*_) was employed to quantify the contamination from hazardous compounds [38,61]. It serves as an effective tool for monitoring pollution over a period of time [55]. The *C*_*f*_ value for each element was calculated using the following equation:


$$C_{f}=\frac{C_{m}}{C_{b}}$$ (2)

Where *C*_*m*_ is the concentration of the element in the collected samples, *C*_*b*_ is the background value of the specific metal in the native soil. According to Buat-Menard and Chesselet (1979), when *C*_*f *_< 1: low pollution factor; 1 ≤ *C*_*f *_< 3: moderate pollution factor; 3 ≤ *C*_*f*_ < 6: considerable pollution factor; *C*_*f *_≥ 6: very high pollution factor [62].

### Degree of contamination

The degree of contamination (*Cdeg*) is the sum of the pollution factors for the investigated elements, and it was calculated using the following equation [38].


$$C_{deg}=\sum_{i=\text{1}}^{i=n}C_{f}$$ (3)

Where the following classification was proposed: *Cdeg *< 6: indicates a low degree of contamination; 6 < *Cdeg* < 12: represents a moderate degree of contamination; 12 < *Cdeg* < 24 signifies a considerable degree of contamination; and *Cdeg* > 24: indicates a high degree of contamination, suggesting severe anthropogenic pollution.

### Modified contamination degree (*mCdeg*)

The modified contamination degree was introduced to estimate the overall contamination level at a site [63]. The *mCdeg* was calculated using the following equation:


$$mC_{deg}=\frac{\sum_{i=\text{1}}^{i=n}C_{f}}{n}$$ (4)

Where *n* is the number of elements analyzed, *mCdeg* < 1.5: null to very low degree of contamination; 1.5 < *mCdeg *< 2: low degree of contamination; 2 < *mCdeg* < 4: moderate degree of contamination; 4 < *mCdeg* < 8: high degree of contamination; 8 < *mCdeg *< 16: very high degree of contamination; 16 < *mCdeg* < 32: extremely high degree of contamination; *mCdeg* ≥ 32: ultra-high degree of contamination.

### Pollution load index

The Pollution Load Index (*PLI*) integrates multiple pollutant factors to determine the contamination potential, representing the relationship between the production level and the pollutant load of each element concerning the total number of parameters analyzed [64,65]. The pollution load index was calculated from the values of the pollution factor of individual elements using the following equation.


$$PLI=\sqrt[{n}]{Cf\text{1}\times Cf\text{2}\times \ldots Cfn}$$ (5)

Where *n* is the number of metals evaluated, Cfn is the pollution factor of a specific metal. *PLI *< 1: uncontaminated; *PLI* = 1: slightly contaminated; *PLI* > 1: environment deterioration.

### Assessment of ecological risk

The potential ecological risk index was proposed by Hakanson (1980) to evaluate the characteristics and environmental behavior of metal contaminants [38].

#### Ecological risk factor (*Eri).*

The primary function of this index is to indicate the contaminating agents and prioritize pollution studies. The ecological risk factor (*Eri*) is calculated using the following equation, with $C_{f}$ representing the pollution factor and *T*_*ri*_ representing the toxic response factor, which indicates the potential hazard posed by specific metals, considering their toxicity and the environmental sensitivity to contamination. According to the standardized toxic response factor (*T*_*ri*_) proposed by Hakanson (1980), Hg, Cd, As, Co, Cu, Pb, Cr, and Zn have toxic response factors of 40, 30, 10, 5, 5, 5, 2, 1, respectively.


$$Eri=T_{ri}xC_{f}$$ (6)

Where *Eri* < 40: low ecological risk; 40 < *Eri *< 80: moderate ecological risk; 80 < *Eri *< 160: considerable ecological risk; 160 < *Eri *< 320: high ecological risk; *Eri *> 320: very high ecological risk.

#### Potential ecological risk index (*IR*).

The potential ecological risk index in the soil was associated with the level of contamination, defined as the sum of individual ecological risk factors. This method comprehensively evaluates the synergy, toxicity, concentration, and ecological sensitivity of the analyzed elements [39]. The risk index for all measured metals was calculated using the following equation.


IR=∑i=1nEri (7)

Where for the potential ecological risk index, the following terminology was utilized is *IR* < 150: Low ecological risk or low ecological contamination level; 150 ≤ *IR *< 300: Moderate ecological contamination level or moderate ecological risk; 300 ≤ *IR *< 600: Considerable ecological risk or serious ecological contamination level; and *IR *> 600: Very high ecological risk or severe ecological contamination level [38,45].

### Statistical analysis

To evaluate the concentrations of potentially toxic elements (PTEs) in mining environmental liabilities (MELs), the Shapiro–Wilk test was first applied to assess the normality of the data. When the assumption of normality was met, a one-way analysis of variance (ANOVA) was conducted to compare the means among the study areas, followed by Tukey’s post hoc test to identify significant differences. In cases where the data did not meet the normality assumption, the non-parametric Kruskal–Wallis test was applied, followed by Dunn’s test for multiple comparisons. All statistical analyses were performed at a 95% confidence level (α = 0.05).

Furthermore, Principal Component Analysis (PCA) was conducted to explore the relationships between metals and their distribution across the different areas, facilitating an understanding of the variability in the collected data. Spearman’s correlation analysis was employed to investigate the relationships between PTEs within MELs.

All statistical analyses were performed using RStudio version 4.0.3 and QGIS software version 3.32.

## Results

### Characterization of the area and environmental liabilities

The study area characterization is summarized in Table 1, which includes observations on vegetation, rural communities, water bodies, and human activity near the sampling sites. Shrub, scrub, and grassland vegetation were observed in the vicinity of the mining environmental liabilities (MELs). Evidence of wildlife was also recorded, including small mammals, camelids, and wildcats, as well as domestic livestock corrals associated with pastoral activities of the local communities.

**Table 1:** Characterization of environmental liabilities.

MELs	Mining Extraction	Coordinates (Altitude)	Residue Types	Water bodies	Vegetation	RC or PC*	Wildlife signs	Livestock	People access	pH
**Chulluncane** **District: Palca**	Cu	19k 0408217 8033184 (3941 m.s.n.m.)	Waste rock and tailings	Caplina River at a distance of about 20 m.	At a distance of 5 meters, scrub-type flora was observed.	Yes	Yes (Rodent feces were found inside the mine openings)	Sheep pens (300 m) and feces were observed.	No	4.98
**Cercana** **District: Pachía**	Cu	19k 0390208 8027180 (1981 m.s.n.m)	Waste rock and tailings	Caplina River at a distance of about 2 m.	At a distance of 1 meter, shrub-like flora was observed.	Yes	No	No	Yes (Farmers within 200 m)	7.41
**Tutupaca** **District: Candarave**	S	19k 0358602 8113940 (4687 m.s.n.m)	Waste rock	Not presented	On the residues, there was scrub-type flora.	Yes	Yes (Camelid and wild cat tracks were seen at a distance of 20 and 50 m, respectively)	No	No	2.13
**Yucamane** **District: Candarave**	S	19k 0362832 8103048 (4033 m.s.n.m)	Waste rocks	Callazas River at a distance of about 5 m.	At a distance of 1 meter, grassland-like flora was observed.	Yes	Yes (Traces of wild cats were seen on the waste)	Sheep feces were seen on the waste.	No	< 1
**Cano** **District: Susapaya**	S	19k 0388911 8090115 (4231 m.s.n.m)	Waste rocks	Not presented	On the residues, there was scrub-type flora.	No	Yes (Camelid tracks in the residue).	Camelid feces were observed.	Yes (Population a 3 meters)	< 1

*RC: Rural Communities, PC: Population Centers. Cu: Copper, S: Sulfur.

In the case of pH, values exhibited considerable variability among the sites, with extremely acidic conditions (< 2.1) recorded at Tutupaca, Yucamane, and Cano, while neutral conditions (pH 7.41) were observed at Cercana.

**Fig 2:**
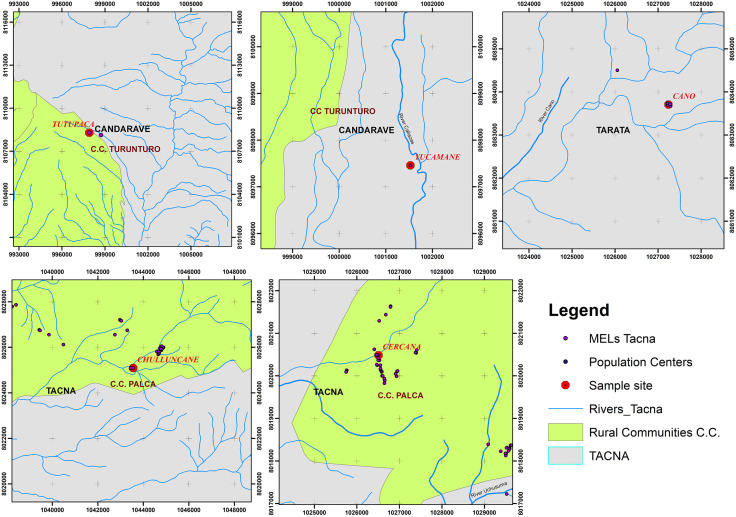
Localization of rural communities near environmental liabilities and bodies of water. Sample site: Tutupaca, Yucamane, Cano, Chulluncane, Cercana. Rural Communites: C.C. Turunturo and C.C. Palca.

A critical finding was the immediate proximity of the sampling points to the region’s main river systems (Caplina, Callazas, Yungani, and Cano), which serve as essential water sources for both rural communities and local ecosystems. Particularly concerning is the location of human settlements, such as Turunturo and Palca, near areas affected by mining environmental liabilities (MELs) (Fig 2). This situation substantially increases the risk of contaminant exposure for surrounding ecosystems and populations, creating a complex environmental risk scenario that demands urgent and site-specific interventions.

This work is particularly relevant since many mining environmental liabilities are located in remote areas with limited accessibility, which hinders their proper characterization by governmental authorities. In most cases, these areas remain abandoned, lack legally identifiable responsible parties, and are not subject to comprehensive contaminant assessments. Based on the findings of this study, a clear environmental risk is associated with the identified contamination, and in the absence of direct responsible parties, it is the responsibility of the State to undertake the remediation and proper characterization of these sites to ensure the protection of ecosystems and the safety of local communities.

### Soil Contamination by Potentially Toxic Elements (PTEs)

The results reveal alarming levels of contamination by potentially toxic elements (PTEs) in soils affected by mining environmental liabilities (MELs) (Table 2), with significantly elevated concentrations (mean ± standard deviation) of at least one contaminant in the studied zones (S1–S5) compared to the reference soil (S6). The concentrations recorded at sites impacted by MELs significantly exceeded both the background soil values (TEL) and the permissible limits established by Peruvian environmental regulations (PEL). At the Cercana site, the concentrations of Pb, As, and Cd exceeded the permissible limits by factors of 19, 7, and 11, respectively. Similarly, at Tutupaca and Cano, free cyanide concentrations surpassed the regulatory thresholds by up to eightfold.

**Table 2:** Concentration of Potentially Toxic Elements (mg/kg) in the environmental liabilities.

MELs Site	Type	Arsenic (mg/kg)	Barium (mg/kg)	Cadmium (mg/kg)	Chromium (mg/kg)	Mercury (mg/kg)	Lead (mg/kg)	Free Cyanide (mg/kg)	Hexavalent Chromium (mg/kg)
**Chulluncane**	MEL	1,102.67 ± 665.64^**^	111.08 ± 94.04^ab^	27.83 ± 29.35^**^	8.81 ± 0.68^**^	3.03 ± 3.58^*^	620.33 ± 282.05^*^	0.04 ± 0^**^	0.03 ± 0 ^ns^
**Cercana**	MEL	1009.67 ± 97.18^**^	71.47 ± 52.28 ^a^	271.63 ± 337.67^**^	6.14 ± 2.09^**^	15.26 ± 13.4 ^*^	15,961.87 ± 13,953.11^*^	0.04 ± 0^**^	0.03 ± 0 ^ns^
**Tutupaca**	MEL	5.15 ± 2.59^**^	220.33 ± 66.98 ^b^	0.02 ± 0^**^	3.50 ± 1.72^**^	3.03 ± 4.42^*^	7.27 ± 2.01^*^	19.25 ± 16.73^**^	0.03 ± 0 ^ns^
**Yucamane**	MEL	13.02 ± 9.90^**^	175.33 ± 40.77^ab^	0.02 ± 0^**^	1.42 ± 0.63^**^	4.00 ± 1.33^*^	7.28 ± 1.55^*^	38.53 ± 13.82 ^**^	0.03 ± 0 ^ns^
**Cano**	MEL	1.21 ± 0.80^**^	154.00 ± 14.11 ^ab^	0.02 ± 0^**^	0.76 ± 0.54^**^	0.82 ± 0.14^*^	7.53 ± 7.64^*^	64.03 ± 24.64 ^**^	0.03 ± 0 ^ns^
**Native Soil – TEL**	Native Soil	83.18 ± 77.7^**^	130.20 ± 23.65^ab^	0.25 ± 0.21^**^	11.71 ± 3.03^**^	0.03 ± 0^*^	18.34 ± 15.63	0.05 ± 0^**^	0.04 ± 0 ^ns^
**Peru**^**x**^ **- PEL**	Industrial soil	140.00	2000.00	22.00	1000.00	24.00	800.00	8.00	1.40
**Ecuador** ^**x**^	Soil quality criteria	5	200	0.5	20	0.1	25	0.25	2.5
	Remediation criteria (commercial & industrial only)	15	2000	10	90	10	150	8	1.4
**Brazil** ^**x**^	Industrial	150	7300	160	400	7	4400	–	10
	Reference value for quality	3.5	75	<0.5	40	0.05	17	–	–
	Prevention Value	15	120	1.3	75	0.5	72	–	–
**Argentina** ^**x**^	Soil quality guideline levels	50	2000	20	800	20	1000	100	–
**USA** ^**x**^	SSL Development	5.0E-02	2.00E + 00	5.00E-03	1.00E-01	2.00E-03	–	2.0E-01	1.00E-01

The descending order of PTE concentrations by site was as follows: Chulluncane: As> Pb > Ba > Cd > Cr > Hg > CN⁻ > Cr(VI); Cercana: Pb> As> Cd > Ba > Hg > Cr > CN⁻ > Cr(VI); Tutupaca: Ba > CN⁻ > Pb> As> Cr > Hg > Cr(VI)> Cd; Yucamane: Ba > CN⁻> As> Pb > Hg > Cr > Cr(VI)> Cd; Cano: Ba > CN⁻ > Pb> As> Hg > Cr > Cr(VI)> Cd.

**Fig 3:**
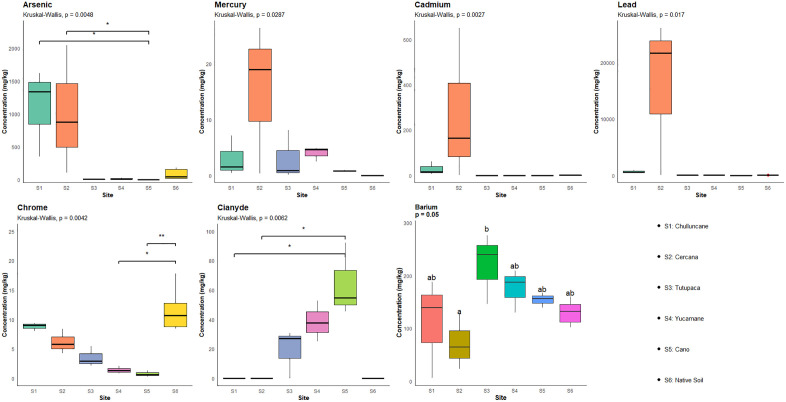
The concentration of elements analyzed for each MELs. S1: Chulluncane, S2: Cercana, S3: Tutupaca, S4; Yucamane, S5: Cano, S6: Reference site (Native soil).

The comparative analysis among the evaluated sites (Table 2 and Fig 3) revealed statistically significant differences in the concentrations PTEs (p < 0.05). The results demonstrate clear variations in contamination levels, allowing for the precise identification of the most affected areas. This analytical approach is essential for environmental management, as it enables the prioritization of contamination hotspots and informs the design of more effective remediation strategies.

The inclusion of a reference site, or native soil, characterized by low contaminant concentrations, strengthens the statistical interpretation by providing a baseline against which anthropogenic contamination levels can be assessed, thereby distinguishing natural background conditions from pollution caused by human activities.

Extremely acidic conditions (pH < 2.1) were observed at Chulluncane, Tutupaca, Yucamane, and Cano, which enhance the mobility and bioavailability of heavy metals. Although the concentrations of total chromium (Cr) and its hexavalent form (Cr(VI)) remained below the thresholds established by national regulations, their presence in a highly acidic environment may increase their potential for dispersion and accumulation in the ecosystem.

For datasets with normal distribution, a one-way ANOVA followed by Tukey’s post hoc test was applied at a 95% confidence level (different letters indicate significant differences). For data that did not meet the assumption of normality, the Kruskal–Wallis test followed by Dunn’s post hoc test was used at the same confidence level.

Significance levels: ns = not significant; * = p < 0.05 (significant); ** = p < 0.01 (highly significant). Statistical tests used: Kruskal–Wallis for Arsenic, Cadmium, Chromium, Mercury, Lead, and Free cyanide; ANOVA for Barium.

Peru^x^: Limits established by Supreme Decree No. 011–2017; Ecuador^x^: Executive Decree No. 3516; Brazil^x^: CETESB Board Decision No 125/2021/E; Argentina^x^: Decree No. 831/93 – Regulation of Law No. 24.051; USA^x^: EPA/540/R/99/005.

The toxic elements associated with mining environmental liabilities reported in this study represent a source of pollution that raises concerns within the community due to the risks they pose to the ecosystem. In Table 3, presents a compilation of international studies that report on sampling locations, types of mining activities, and the specific contaminant metals investigated, in a manner comparable to the approach used in this study.

**Table 3:** Soil contaminants from mining activities.

Country	City/Region	Sampling location	Mining Extraction Type	Pollutants	Reference
**Peru**	Tacna	Soils Contaminated by Mining Environmental Liabilities	Cu, S	As, Cd, Pb, Hg, CN^-^	This study
**Peru**	Cajamarca	Soils Contaminated by Mining Environmental Liabilities	–	As, Ag, Cd, Cu, Pb, and Zn	[11]
**China**	Daye	Actividades de fundición	Cu	Cd, Cu, Pb, As	[66]
**Spain**	Madrid	Abandoned Monica mine	Minerals (arsenopyrite, matildite and sphalerite)	As, Cu, Zn, Cd, Pb, W, Ag, Fe	[31]
**Mexico**	Santa Maria de la Paz	Mining Area	Pb-Zn-Ag (Cu-Au) skarn ore system	As, Cu, Pb y Zn,	[67]
**Ghana**	Tarkwa, Ashanti, Atewa	Mining areas	Au	As, Cd, Hg, Zn, Co, Cu, Mn, Fe, Al, V, Cr, Pb	[54]
**China**	Hunan,	Nonferrous metal mine	Zn, Pb	Zn, Pb, Cd, Cu, As	[39,68]
**Turkey**	Kutahya	Contaminated soils by mining area	Ag	As, Ag, Pb	[69]
**Venezuela**	Bolívar	Soils Of The Mining Sector	Au	Hg	[70]
**Kosovo**	K. Mitrovica	soil of mining centres	Pb, Zn, As, Cd	As	[71]
**Colombia**	In northern Colombia	Soils Contaminated with Mining Activities	Au	Hg, Pb y Cd	[72]
**Slovakia**	Central Spiš	Soil Heavy Metal Pollution	Cu, Hg	Hg, Zn y Cd	[73]
**India**	Karnataka	Tailings	Au	Cd, Cu, Pb, Zn	[2,74]
**Poland**	Zloty Stok	Mining soil	Au, As	As	[75]
**Peru**	Pasco	soil in mining towns	Ag, Au	Pb	[76]
**Kosovo**	Mitrovica	Soil and tailing	Pb, Cd, Zn	Pb	[77]
**Colombia**	Sabana de Bogotá	Soil	–	Pb	[78]
**Cyprus**	Lefke	Soil and plant	Cu	Fe, Zn, Pb, Cd, Cu, Ni, Cr	[79]
**Mexico**	San Francisco of Oro (Chihuahua)	Soil	–	Pb, Zn, Cd, As	[80]
**Ethiopia**	Adola	Sediment, water and plant	Au	Al, Mn, Fe, As, Ni, Cr, Cu, Co, Pb, W, Sb, Mo, Zn, V	[53]
**Poland**	Tarnobrzeg County	Mining Soil Substrate	S	N, Ca, Mg, Al, S	[81]
**China**	Hainan	Soils, mine tailing, and groundwater samples	Au	Pb, As, Cd, Hg, CN^-^	[26]

### Environmental indices

#### Geoaccumulation index.

Calculations of the Geoaccumulation Index indicate elevated contamination levels at several Mining Environmental Liability (MEL) sites Table 4. Specifically, the Chulluncane MEL exhibits extreme contamination levels for Cd and Hg (5.235 and 5.229, respectively) and strong contamination levels for As and Pb (3.807 and 3.122). The Cercana MEL shows extreme contamination levels for Hg, Pb, and Cd (7.011, 6.585, and 5.713, respectively), as well as high to extreme concentrations of As (4.301).

**Table 4:** The study includes the Geoaccumulation Index of environmental liabilities.

Pollutant	Geoaccumulation Index				
	Chulluncane	Cercana	Tutupaca	Yucamane	Cano
**As**	3.807	4.301	−2.583	−4.632	−7.777
**Ba**	−2.135	−1.414	0.341	−0.339	−0.367
**Cd**	**5.235**	**5.713**	−2.392	−2.755	−3.832
**Cr**	−1.603	−1.701	−2.028	−3.601	−4.348
**Hg**	**5.2289**	**7.011**	4.445	**6.411**	4.174
**Pb**	3.122	**6.585**	−0.682	−1.055	−1.191
**CN** ^**-**^	−0.907	−0.907	**5.421**	8.94	**9.671**
**Cr(VI)**	−1	−1	−1	−1	−1

≤ 0, Unpolluted; 0 ≤ *I-geo* ≤ 1, Unpolluted to moderately polluted; 1 ≤ *I-geo* ≤ 2, Moderately polluted; 2 < *I-geo* ≤ 3, Moderately to strongly polluted; 3 ≤ *I-geo* ≤ 4, Strongly polluted; 4 ≤ *I-geo* ≤ 5, Strongly to extremely polluted; and 5 < *I-geo*, Extremely high polluted.

In Tutupaca, contamination is extreme only for free CN⁻ (5.421), while Hg contamination is at high to extreme levels (4.445). The Yucamane MEL exhibits extreme contamination levels for CN⁻ and Hg (8.94 and 6.411, respectively), while the Cano MEL shows extreme contamination for free CN⁻ (9.671) and high to extreme contamination levels for Hg (4.174).

This analysis underscores the severity of contamination in several locations, highlighting the urgent need to implement mitigation measures and establish continuous monitoring to reduce environmental impacts in these areas.

### Contamination factor

The results obtained from the Contamination Index (C_*f*_), shown in Table 5, provide a framework to understand and quantify the presence of hazardous compounds in the environment caused by Mining Environmental Liabilities (MELs). According to the analysis, sites with C_*f*_ values exceeding 6 indicate a high level of contamination.

**Table 5:** Contamination Factor Index of Environmental Liabilities Included in the Study.

Pollutant	Pollution Factor				
	Chulluncane	Cercana	Tutupaca	Yucamane	Cano
**As**	**25.367**	**52.057**	0.271	0.072	0.008
**Ba**	0.694	0.701	1.967	1.209	1.167
**Cd**	**84.333**	**468.328**	0.286	0.222	0.105
**Cr**	0.495	0.480	0.397	0.13	0.090
**Hg**	**100.856**	**508.778**	**101.156**	**133.444**	**27.333**
**Pb**	**14.003**	**732.196**	0.957	0.734	0.932
**CN** ^**-**^	0.800	0.800	**384.933**	**770.667**	**1,280.667**
**Cr(VI)**	0.750	0.750	0.750	0.750	0.750

*C*_*f*_ < 1: low; 1 ≤ *C*_*f*_ < 3: moderately; 3 ≤ *C*_*f*_ < 6: considerably; *C*_*f*_ ≥ 6, Very high.

The contamination levels were ranked in descending order for each site. In Chulluncane, the order is Hg > Cd> As> Pb. In Cercana, Pb > Hg > Cd> As. In Tutupaca, CN⁻ > Hg. In Yucamane, CN⁻ > Hg. Finally, in the MEL located in Cano, CN⁻ > Hg. The predominant contaminants in each MEL are Hg, Pb, and CN ⁻ , which are particularly significant in terms of environmental impact.

High C_f_ values emphasize the substantial influence of anthropogenic contaminants, whereas lower values reflect the geological distribution of soil components, underscoring the need for proper monitoring and management in these areas.

### Pollution load index

**Fig 4:**
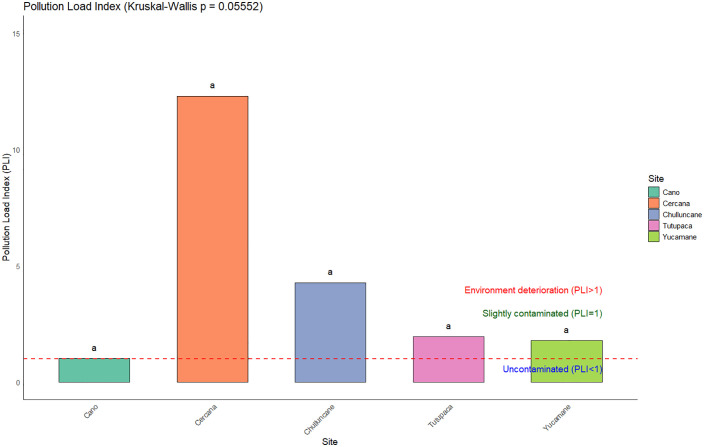
Pollutant Load Index (*PLI*) for five sites. A PLI value greater than 1 indicates environmental deterioration. The red dashed line denotes the upper threshold established according to the interpretative scales of the index. The letter ‘a’ indicates no significant differences between sites (Kruskal–Wallis test with Dunn’s post hoc analysis).

The Pollution Load Index (PLI) values obtained for each evaluated site (Fig 4) reflect the levels of contamination by potentially toxic elements (PTEs), in the following descending order: Cercana (12.3)> Chulluncane (4.3)> Yucamane (1.8)> Tutupaca (1.95)> Cano (1.02). The overall average across all evaluated areas was 4.3. All sites exhibit PLI values greater than 1, indicating the presence of contamination likely associated with mining environmental liabilities (MELs). These values may be related to persistent mineral extraction processes, which contribute to the increase in environmental pollution.

Although some sites exhibit considerably high PLI values, statistical analysis did not reveal significant differences among them (p = 0.05552), as the result was slightly above the 5% significance threshold. Nevertheless, the Cercana site stands out as a critical hotspot due to its elevated contamination index, underscoring the need for complementary assessments and the implementation of targeted remediation measures, even in the absence of statistical significance.

### Degree of contamination, modified degree of contamination, and potential ecological risk

The environmental indices applied (*Cdeg* and *mCdeg*), along with the ecological risk index (Er), provided an integrated assessment of contamination intensity and the ecological risk associated with potentially toxic elements (PTEs) at the evaluated sites. These values were compared across sites and classified according to categorical thresholds proposed in the literature, which facilitated the interpretation of contamination and ecological risk levels.

According to the results of *Cdeg* and *mCdeg*, all sites exceeded the upper limits defined by the interpretative scales, indicating contamination by multiple PTEs. The highest *Cdeg* value was observed at Cercana (2,640), followed by Cano (1,311), Yucamane (907), Tutupaca (490), and Chulluncane (227). The *mCdeg* index, which minimizes the influence of extreme values and offers a more stable assessment, confirmed this pattern. Cercana again exhibited the highest value (330), followed by Cano (163), Yucamane (113), Tutupaca (68), and Chulluncane (28). In all cases, both *Cdeg* and *mCdeg* values far exceeded the maximum thresholds of their respective interpretative scales (24 and 32, respectively).

**Fig 5:**
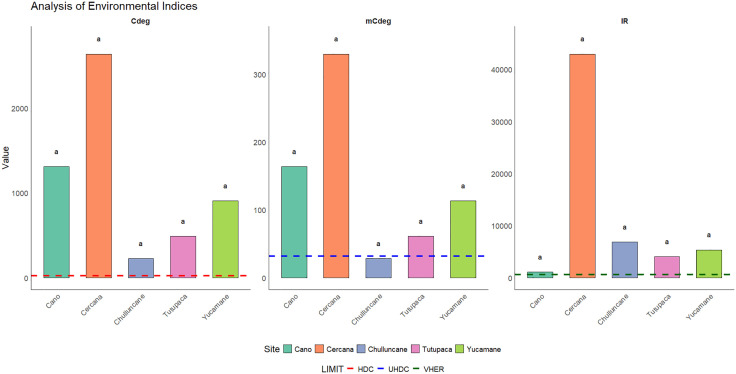
Potential ecological risk. The figure illustrates the Degree of Contamination (*Cdeg*), the Modified Degree of Contamination (*mCdeg*), and the Ecological Risk Index (IR). The dotted lines indicate the upper limits established by the interpretative scales for each index: HDC > 24 (high degree of contamination, red line), UHDC ≥ 32 (ultra-high degree of contamination, blue line), and VHER > 600 (very high ecological risk, green line). Statistical analysis was performed using the Kruskal–Wallis test followed by Dunn’s post hoc test. No statistically significant differences were found among the sites (p > 0.05); different letters would indicate significant differences.

Regarding potential ecological risk (Fig 5), all sites exceeded the established maximum value (600), placing them within the very high ecological risk category. This indicates severe contamination with a significant latent risk, primarily driven by anthropogenic sources of As, Ba, Cd, Hg, Pb, and CN ⁻ .

Although notable differences in contamination levels were observed among the sites, statistical analysis did not reveal significant differences (p > 0.05), possibly due to internal variability. Nevertheless, the magnitude of the recorded indices supports the urgent need for site-specific remediation measures for these mining environmental liabilities.

### Principal component analysis (PCA) of EPT and site distributions

**Fig 6:**
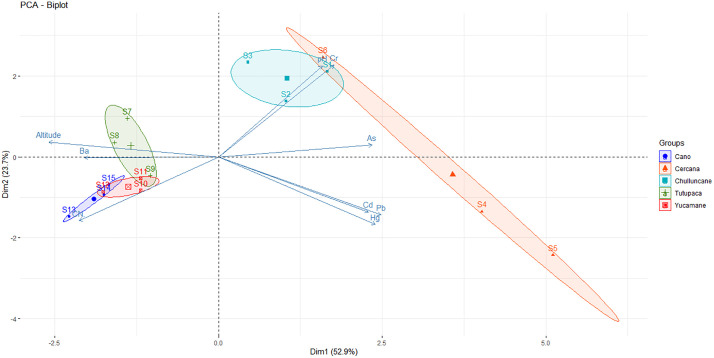
Principal Component Analysis (PCA) graphs of EPT concentrations (As, Cd, Cr, Pb, Br, Hg, and CN^-^) and their spatial distribution in environmental sites of S1: Cercana, S2: Chulluncane, S3: Tutupaca, S4: Yucamane, S5: Cano. Circular lines on the PCA graphs overlap to indicate sampling sites in the same area.

The first two axes of the PCA accounted for 76.6% of the total variance (PC1: 52.9%, PC2: 23.7%), reflecting the relationship between environmental variables, specifically potentially toxic elements (PTEs) and altitude, and the sampling sites associated with mining environmental liabilities, grouped into five zones: Cano, Cercana, Chulluncane, Tutupaca, and Yucamane (Fig 6).

The sites in Cercana (S4–S6) were strongly associated with elevated concentrations of Cr, Pb, and As, while Yucamane (S10–S12) showed a moderate association with Hg and Cd, suggesting a direct influence of mining liabilities in both areas. In contrast, Tutupaca (S7–S9) aligned primarily with altitude and Ba, and Cano (S13–S15) was related to CN ⁻ , indicating distinct geochemical conditions compared to the other sites. Chulluncane (S1–S3) occupied an intermediate position, without a strong association with any particular PTE. This analysis reveals distinct geochemical patterns in the distribution of contaminants and highlights the utility of PCA for identifying geochemical variability and assessing the environmental impact of mining liabilities, thereby supporting more effective management and monitoring strategies.

### Correlation between parameters

**Fig 7:**
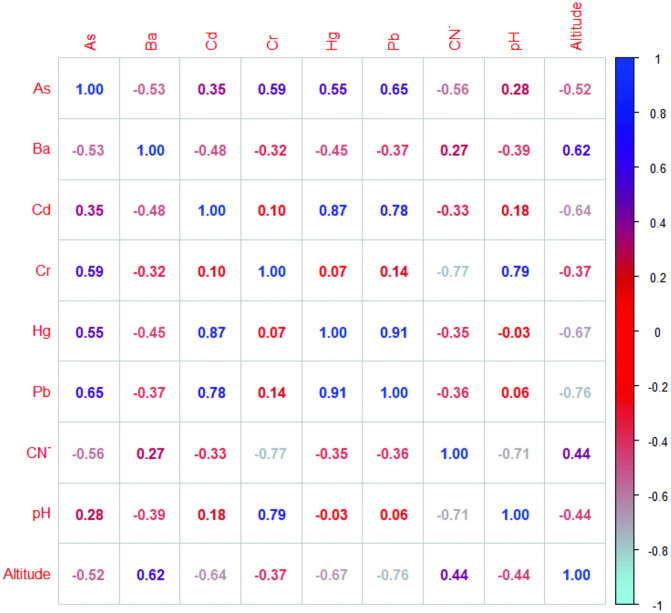
Spearman rank order correlation for selected parameters analyzed in soil samples from mining environmental liabilities.

The Spearman correlation analysis reveals significant relationships between potentially toxic elements, pH, and altitude, offering key insights into environmental processes and the distribution of contaminants in the evaluated areas (Fig 7). Strong positive correlations were observed between Cd/Hg (r = 0.87), Cd/Pb (r = 0.78), Hg/Pb (r = 0.91), CN ⁻ /Cr (r = −0.77), As/Cr (r = 0.59), As/Hg (r = 0.55), and CN ⁻ /pH (r = 0.71). These associations suggest a shared contamination source, likely linked to mining environmental liabilities, as well as similar environmental processes governing their distribution. In addition, the positive correlation between Cr and pH (r = 0.79) indicates that less acidic conditions may reduce chromium mobility in soils, thereby limiting its dispersion. Negative correlations were also relevant. The strong inverse relationship between CN⁻ and Cr (−0.77) reflects differences in geochemical behavior or origin. Likewise, the negative correlations of Pb (−0.76) and Hg (−0.67) with altitude, reflecting that the concentrations of these metals decrease in higher-altitude zones, possibly due to topographic and climatic factors that limit their deposition or transport.

Overall, these correlations highlight the interaction between chemical (pH) and geographic (altitude) factors and the concentrations of potentially toxic elements in mining environmental liabilities. The co-occurrence of elements such as Cd, Hg, and Pb supports the interpretation of a common anthropogenic origin, likely derived from similar pollution sources associated with past mining activities.

## Discussion

The mining environmental liabilities (MELs) evaluated in this study represent a critical threat due to the presence of potentially toxic elements (PTEs), primarily Pb, As, Cd, and CN ⁻ . The proximity of these sites to water bodies, rural communities, and agricultural and livestock activities increases the risk of contaminant dispersion into surrounding ecosystems [27,66,82]. This situation compromises ecological stability, affecting hydrological systems such as the Caplina, Callazas, Yungani, and Cano rivers, as well as vulnerable populations, including Turunturo and Palca [82,83]. These conditions create a scenario of high environmental vulnerability, especially in areas with limited access to drinking water and healthcare services. Furthermore, surface runoff and infiltration facilitate the transport of contaminants, amplifying their impact on ecosystem functionality and public health [82,84]. The detection of extremely acidic pH levels (pH < 2.1) in sites such as Tutupaca, Yucamane, and Cano confirms the persistence of acid mine drainage processes [29,30], a condition that promotes the solubility, mobility, and bioavailability [26]. Although total chromium and Cr(VI) concentrations did not exceed regulatory thresholds, their coexistence with acidic conditions suggests a potential risk of toxicity and accumulation across ecosystem compartments.

Historically, the absence of environmental regulations and restoration programs has facilitated the accumulation of MELs in Peru [9,13,19]. This situation is exacerbated by the high concentrations of PTEs, their persistent toxicity, the lack of detailed studies in remote areas, and the limited implementation of remediation strategies [21,26,85].

The average concentrations of PTEs in most evaluated sites exceeded TEL and PEL values, indicating severe multielement contamination in degraded mining environments [26]. The heterogeneous distribution of contaminants such as As, Pb, Ba, Cd, Cr, Hg, CN ⁻ , and Cr(VI), with significant differences between sites (p < 0.05), suggests a combined influence of substrate mineralogy and the specific extractive practices employed during extraction, milling, and smelting [86]. Our findings are consistent with studies conducted in the Peruvian Andes, where markedly elevated concentrations of Pb, Zn, As, Cu, Ag, and Cd were reported in areas impacted by MELs [1]. Additionally, studies in abandoned mines have identified the presence of As, Cd, Cr, Co, Cu, Pb, Mn, Ni, and Zn, with distributions influenced by both geological factors and anthropogenic sources [56,87]. Acid drainage has also been documented in inactive mining sites, with the release of metals such as Al, Ca, Co, Cu, Fe, Mg, Mn, Ni, and Zn, mainly accumulating due to exposed waste that can act as geochemical barriers and alter migration pathways [29]. In particular, arsenic can cause severe health effects, including carcinogenic risks [25,32,88]. The presence of As is often associated with copper and gold-copper deposits. Elevated concentrations have been reported in abandoned mines and tailings areas [89]. For instance, in Salamanca (Spain), extreme levels of As (>1,000 mg/kg) have been attributed to altered granites or metamorphic rocks containing sulfides [67,90]. In ancient mining zones, concentrations reached up to 29,000 mg/kg [67]. In Cajamarca, Pb and As exceeded soil quality standards, while Cd remained within permissible limits [11]. In Tacna, our results showed concentrations up to 650 mg/kg for Cd, 2,046 mg/kg for As, and 16,000 mg/kg for Pb, with some samples reaching 26,131 mg/kg of Pb, 650 mg/kg of Cd, and 92 mg/kg of free cyanide, representing severe ecological risks. These findings reinforce the evidence that MELs are persistent contamination hotspots with critical implications for environmental management and ecosystem health [60].

The environmental indices applied in this study (I-geo, C*f*, PLI, *Cdeg*, and *mCdeg*) provide a robust quantitative framework for assessing the magnitude, extent, and ecological implications of contamination associated with MELs. The combined analysis of I-geo and C*f* revealed critical environmental effects for at least one PTE at all sites, particularly As, Cd, Hg, Pb, and CN ⁻ , with the highest intensities at Cercana and Chulluncane, suggesting a strong anthropogenic influence. The PLI indicated progressive environmental degradation, identifying Cercana (PLI = 12.3) as a critical contamination hotspot. These elevated values reflect the persistence of unresolved contamination sources, reinforcing the role of MELs as major contributors to environmental pollution. The *Cdeg* and *mCdeg* indices confirmed widespread contamination, while the RI index classified all sites as having very high ecological risk [24]. Although no statistically significant differences were found between sites (p > 0.05), the magnitude of the recorded values supports the urgent need for site-specific and prioritized remediation strategies.

Principal Component Analysis (PCA) identified distinct geochemical patterns, with Cercana and Yucamane associated with high concentrations of Cr, Pb, As, Hg, and Cd [60], while Cano showed a strong relationship with CN ⁻ . These trends were consistent with the results of Spearman correlation analysis, which revealed positive associations among Cd, Hg, and Pb, as well as significant relationships between PTEs, pH, and altitude. The positive correlation between Cr and pH suggests lower mobility under neutral conditions, while the negative correlations with altitude indicate reduced concentrations of Pb and Hg at higher elevations, potentially due to topographic or climatic constraints [67,91].

Persistent contamination patterns associated with MELs were identified, posing a severe environmental risk exacerbated by local climatic conditions. Rainfall runoff and nearby water bodies may facilitate contaminant transport toward adjacent communities such as Turunturo and Palca, increasing exposure to mining residues [82,84]. The potential entry of PTEs into water bodies [13,26,92], agricultural soils, and crops [22], represents a serious threat to ecosystems [93], compromising biodiversity and posing health risks through ingestion, inhalation, and dermal contact [87,88,94]. Moreover, carcinogenic effects on lungs, liver, kidneys, bladder, and breast tissue have been documented, along with cardiopulmonary disorders and gene expression alterations [87,88,95,96]. Chronic exposure has also been linked to diseases such as Alzheimer’s, osteoporosis, diarrhea, and vomiting [6], as well as social impacts on vulnerable communities [3,25,80].

These findings reaffirm the magnitude of the environmental problem associated with MELs in high Andean regions and highlight the consequences of the historical absence of environmental regulation and management. The methodological convergence of multiple indices and multivariate analyses provides a solid scientific basis for establishing intervention priorities. It is recommended to design and implement long-term environmental remediation, ecological restoration, and systematic monitoring strategies aimed at mitigating the impacts of PTEs on ecosystems and human health [26].

## Conclusions

Mining environmental liabilities (MEL) pose a critical problem due to the extremely high concentrations of arsenic, cadmium, lead, free cyanide, and mercury, which significantly exceed regulatory standards and create an extreme ecological risk to local biota and surrounding areas. Pollution and ecological risk indices (I-geo, C*f*, PLI, *Cdeg*, IR, E*ri*) have highlighted severe environmental impacts, while multivariate analysis revealed multiple contamination sources and the coexistence of toxic elements influenced by similar environmental processes. These conditions, compounded by the proximity to flora, fauna, water bodies, and rural communities, underscore the urgent need for mitigation strategies.

It is imperative to establish continuous monitoring programs, implement sustainable remediation strategies, and develop policies that prioritize reducing the contaminant load and restoring degraded ecosystems. The lack of a situational diagnosis has delayed mitigation actions, leaving these liabilities unaddressed. Globally, MEL represents a significant environmental challenge, demanding the adoption of international management standards and sustainable technological strategies to balance mining activity with environmental conservation and sustainable development.

## Supporting information

S1 Table Concentrations of Heavy Metals in Soils from Mining Environmental Liabilities and Native Soils. (XLSX)
